# Trophic Interactions Between Two Herbivorous Insects, *Galerucella calmariensis* and *Myzus lythri*, Feeding on Purple Loosestrife, *Lythrum salicaria*, and Two Insect Predators, *Harmonia axyridis* and *Chrysoperla carnea*

**DOI:** 10.1673/031.007.3001

**Published:** 2007-05-10

**Authors:** Bethzayda Matos, John J. Obrycki

**Affiliations:** ^1^ Department of Entomology, University of Kentucky, Lexington KY 40546; Department of Entomology, Iowa State University, Ames IA 50011

**Keywords:** trophic cascade, biological control of weeds, predator-prey interactions

## Abstract

The effects of two herbivorous insects, *Galerucella calmariensis* Duftschmid and *Myzus lythri* L. (Coleoptera: Chrysomelidae), feeding on purple loosestrife, *Lythrum salicaria* L. (Myrtiflorae: Lythraceae), were measured in the presence of two insect predators, *Harmonia axyridis* Pallas (Coleoptera: Coccinellidae) and *Chrysoperla carnea* (Stephens) (Neuroptera: Chrysopidae). A greenhouse cage experiment examined the direct effects of these predators on these herbivores, and indirect effects of predation on aboveground biomass, defoliation, number of leaves, and internode length. Eight treatment combinations with *G*. *calmariensis*, *M*. *lythri*, *H*. *axyridis* and *C. carnea* were applied to caged *L*. *salicaria.* The experiment ended when *G*. *calmariensis* adults were observed, 11 to 13 days after release of first instar *G*. *calmariensis. G. calmariensis* larvae alone removed significant amounts of leaf tissue and reduced the number of *L. salicaria* leaves. Predators did not reduce levels of defoliation by *G*. *calmariensis. C. carnea* had no effect on *G*. *calmariensis* survival, but *H. axyridis* reduced *G*. *calmariensis* survival in the presence of *M. lythri.* Both predators reduced the survival of *M*. *lythri.* This short duration greenhouse study did not demonstrate that predator-prey interactions altered herbivore effects on *L. salicaria.*

## Introduction

Purple loostrife, *Lythrum salicaria* L. (Myrtiflorae: Lythraceae) is an invasive weed with high fecundity that displaces native vegetation (Thompson et al. 1987; Balough and Bookhout 1989; Blossey et al. 2001). In the early 1990s, two species of herbivorous insects, *Galerucella calmariensis* L. and *Galerucella pusilla* Duftschmid (Coleoptera: Chrysomelidae), were introduced in North America to reduce *L. salicaria* density (Hight et al. 1995). Since 1994, more than 1.4 million individual *Galerucella* spp. have been released in Iowa wetlands (J.J. Obrycki, unpublished data). In a field cage study, Cortilet (1998) demonstrated that the percentage defoliation and terminal bud damage of *L. salicaria* increased with increasing *G*. *calmariensis* larval density. After 47 days, 50 *G*. *calmariensis* larvae caused 25% defoliation and more than 20 terminal buds per stem were damaged (Cortilet 1998). In a second cage study, ten *L. salicaria* plants were enclosed with 45 *G*. *pusilla* eggs in individual cages for 35 days resulting in 14% defoliation (Wiebe 2001). Additionally, Katovich et al. (1999) released 50 *Galerucella* spp. adults and larvae on caged *L. salicaria* plants for two months, resulting in an average of 86% defoliation.

*Myzus lythri* (Schrank) (Homoptera: Aphididae), first recorded in the U.S. in the 1930s (Gillette and Palmer 1934), was observed feeding on *L. salicaria* in Indiana in 1992 (Voegtlin 1995). In a greenhouse study, significantly lower dry weight of roots and shoots were observed for plants infested with *M. lythri*, compared to plants without aphids (Voegtlin 1995).

Previous studies have shown negative effects on *L. salicaria* when either *M. lythri* (Voegtlin 1995) or *G*. *calmariensis* were the only herbivorous species (Cortilet 1998; Wiebe 2001; Katovich et al. 1999; Landis et al. 2003; Denoth and Myers 2005). However, to our knowledge no studies have examined interactions of *G*. *calmariensis* and *M*. *lythri* on *L. salicaria.* A previous discussion of the potential for biological control of *L. salicaria* predicted that combinations of insect herbivores would have a greater negative effect on *L. salicaria* than single herbivorous species (Malecki et al. 1993). Interspecific interactions of these herbivores on *L. salicaria* allow one to test whether an additive effect reduces *L. salicaria* biomass, or if interspecific competition between the herbivores inhibits reduction of *L. salicaria.* In biological control, interactions are considered when multiple natural enemy species are introduced into a new habitat. Multiple agents are believed to increase cumulative stress on weeds (Myers 1985). Competing insects, located in the same areas of the plant, increase destruction of the plant, thus reducing plant growth (Harris 1981).

*G*. *calmariensis* larvae and *M. lythri* adults and nymphs are suitable prey for preimaginal development of the predatory insects, *Chrysoperla carnea* (Stephens) (Neuroptera: Chrysopidae), and *Harmonia axyridis* Pallas (Coleoptera: Coccinellidae) (Matos and Obrycki 2006). *M. lythri* was highly suitable prey for *H. axyridis* and *C. carnea* (survival of 80% and 61%, respectively), whereas *G*. *calmariensis* was highly suitable prey for *C. carnea* (survival of 76%), but less suitable for *H. axyridis* (survival of 27%) (Matos and Obrycki 2006).

Predator-prey interactions have the potential to be detrimental to suppression of *L. salicaria* through trophic cascades. A trophic cascade occurs when top predators have an indirect influence on the abundance of plant species via their effect on the number of herbivores present (Schmitz et al. 2000). A field study in Sweden, demonstrated that defoliation of *L*. *salicaria* by *G*. *calmariensis* is higher when fewer insect predators are present (Hamback et al. 2000). These results indicated that *G*. *calmariensis* abundance is affected by predation by lady beetles (Hamback et al. 2000). In North American wetlands infested with *L. salicaria,* predation has been reported on *Galerucella* spp. eggs and larvae (Sebolt and Landis 2004; Wiebe and Obrycki 2004).

The objectives of this study were twofold; to determine if two herbivorous species (*G*. *calmariensis* and *M*. *lythri*) have an additive negative effect on selected plant measurements, and, second, whether the presence of insect predators that consume the herbivores reduce the effects of herbivory on *L. salicaria,* causing an indirect effect on the plant.

## Materials and Methods

### Insect cultures

*G*. *calmariensis* adults and *M. lythri* adults and nymphs were reared on *L. salicaria* plants; pea aphids, *Acyrthosiphon pisum* (Harris) (Hemiptera: Aphididae) were reared on fava beans, *Vicia faba* L*.* (Fabaceae). All rearing was done on plants enclosed in cages in the Iowa State University Department of Entomology greenhouse and growth chambers at 25 ± 5°C 16:8 h (L:D). Voucher specimens of *G*. *calmariensis*, *M. lythri*, *C. carnea*, *C. maculata*, and *H. axyridis* were deposited in the Iowa State University Insect Collection, Department of Entomology, Ames, IA.

**Table 1.**
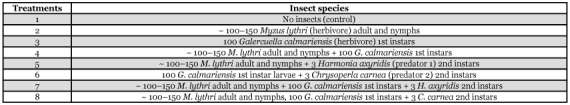
Eight predator-prey combination treatments.

*C. carnea* were purchased from Rincon-Vitova Inc. (Fillmore, CA) as first instars and were fed on a mixture of *Ephestia kuehniella* Zeller (Lepidoptera: Pyralidae) eggs and *A. pisum. H. axyridis* adults were collected from Story County, Iowa. *H. axyridis* adults were maintained in 0.24-liter paper cages (Neptune Paper Products, Jersey City, NJ) in growth chambers (Model No. 1–30 BLL, Percival, www.percival-scientific.com) at 24 ± 1°C with a photoperiod of 16:8 (L:D). Adults were fed pea aphids, *A. pisum*, until females laid eggs, which were collected daily.

### Experimental design

In early June 2004, *L. salicaria* seeds were planted in SunGro Sunshine LC1 Mix® and maintained in a greenhouse at 25 ± 5°C 16:8 h (L:D). When *L. salicaria* plants reached a height of 60 ± 3 cm, each of 42 seedlings were transplanted into single 19-L pots and enclosed in mesh sleeves (120 cm tall × 70 cm wide) (No-See-Um netting, Balsom Hercules Group, Providence, RI) supported by tomato cages.

The experimental design was an incomplete block design with eight treatments and five replicates (Table 1). The marsh habitat of purple loosestrife was simulated using potted plants in each of 10 wading pools (100 cm diameter, 30 cm height, containing 10 cm of water). Seven of the eight treatments were randomly selected and assigned to an individual potted plant in a wading pool, each of which served as a replication. The remaining treatments, which was the eighth pot (totaling five potted plants from the five blocks), were placed in a sixth pool, thereby creating an incomplete block. In the sixth pool, two additional potted plants were placed to create a pool environment similar to the other five pools with seven potted plants.

These treatments were selected because in a previous laboratory study, *G*. *calmariensis* and *M*. *lythri* were found to be suitable prey for the development and survival of *H. axyridis* and *C*. *carnea* (Matos and Obrycki 2006). In addition, due to logistical accommodation of the experimental units, we selected eight treatments out of all possible combinations. This selection was based on two preliminary studies and several prior experimental studies (Voegtlin 1995; Cortilet 1998; Wiebe 2001; Finke and Denno 2004). One plant in each block was randomly assigned to one of eight treatments (Table 1). Predator and herbivore densities were similar to densities observed in the field (Matos and Obrycki 2007 in press). The experiment concluded when *G*. *calmariensis* adults eclosed, because plant measurements were taken in response to *G*. *calmariensis* larval feeding.

### Parameters measured

The number of herbivores and predators in each cage were counted at the end of the experiment. *M. lythri* infested plants for 25 days, *G*. *calmariensis* infested plants for 11–13 days, and predators were caged on plants for 9–11 days. *Myzus lythri* were released on the plants 14–16 days before predators were released and *G*. *calmariensis* first instars were released 2 days before the predators were released.

Five measurements of *L. salicaria* were taken: number of leaves, above ground biomass, internode length, leaf tissue removed, and total leaf area. Above ground dry biomass was determined by harvesting all live aboveground vegetation, which was dried in an oven for 7 days at 55°C and then weighed. Skeletonizing of the leaves occurs as a result of *G*. *calmariensis* larvae feeding. Total leaf area and percentage defoliation (leaf tissue removed) were measured at the end of the experiment using Adobe Photoshop® (version 7.0). Multiple leaves were scanned into a digital format using Hewlett Packard Scanjet 4600 series digital flatbed scanners^®^ (www.hp.com) with HP Photo Imaging Gallery Software (1996–2002) (version 1.1, images scanned at 600 dpi). This technique is similar to O'Neal et al. (2002) who determined that a flatbed scanner is an accurate and precise tool for leaf area measurement. Two copies of the original images were made. To estimate leaf tissue removal, the area removed from a leaf in the copied image was filled with black pixels. The area was measured using the histogram option (selected from the Image menu in Adobe Photoshop^®^).

**Table 2.**
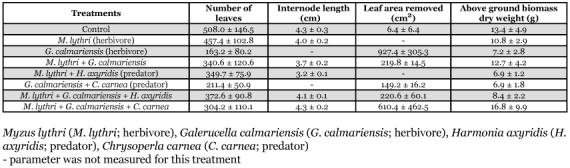
Effects of herbivore-predator treatments on on *Lythrum salicaria*(mean ± SE) in a greenhouse study.

### Data analysis

The herbivore-plant and predator-prey effects on number of leaves, internode lengths, leaf area measured, leaf tissue removal, and aboveground dry biomass of *L. salicaria* were analyzed independently with mixed-model analyses of variance in which a block (replication) was modeled as a random source of variation (SAS 2003). Subsequently, the following contrasts were used to examine the effects of herbivory (control vs. *G*. *calmariensis*, control vs. *M*. *lythri*), interaction of herbivores (control vs. *M*. *lythri* + *G*. *calmariensis*, *G*. *calmariensis* vs. *M*. *lythri* + *G*. *calmariensis*, and *M*. *lythri* vs. *M*. *lythri* + *G*. *calmariensis*), predation on herbivores (*G*. *calmariensis* vs. *C. carnea* + *G*. *calmariensis*, *M. lythri* vs. *H*. *axyridis* + *M. lythri*, *G*. *calmariensis* + *M*. *lythri* vs. *G*. *calmariensis* + *M. lythri* + predator), and predator effect on plant (control vs. herbivores + predator). To determine effects on herbivore survival, means separation was done using LSD statistic test (SAS 2003). Results were considered significant at P < 0.05.

**Tables 3.**
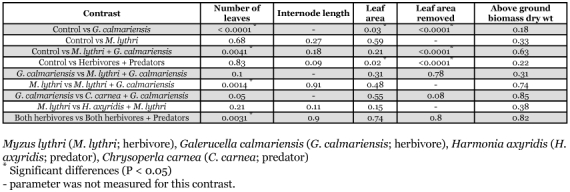
Probability values for single degree-of-freedom linear contrasts of plant parameters.

## Results

### Herbivore - plant interactions

The greatest leaf tissue removal occurred in the *G*. *calmariensis* alone treatment (927.4 ± 305.3 cm^2^; mean ± SE) (Table 2). *G*. *calmariensis* removed a significantly larger amount of leaf tissue when compared to the control (P <0.0001; Table 3). Over 50% defoliation was measured in *L. salicaria* plants with *G*. *calmariensis* only. *G*. *calmariensis* also significantly reduced the number of leaves compared to the control treatments (P < 0.0001; Table 3). *M. lythri* alone did not significantly affect internode length (P = 0.27; Table 3). Plants infested with *M. lythri* had more leaves at the end of the experiment compared to the *M. lythri* + *G. calmariensis* treatment (P = 0.0014; Table 3).

**Table 4.**
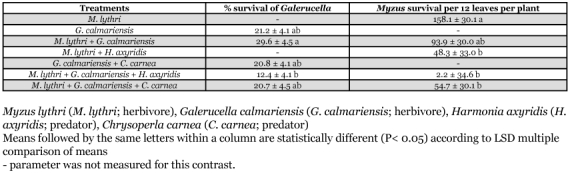
Percentage survival (± SE) of *Galerucella calmariensis* and mean survival (± SE) of *Myzus lythri* alone and in the presence of an individual predator.

### Interactions of two herbivore species

Over 29% of *G. calmariensis* and a mean of 93.9 *M. lythri* per 12 leaves survived in the *M. lythri *plus *G. calmariensis* treatment (Table 4). Total numbers of *G. calmariensis* and *M. lythri* surviving were similar to treatments in which each individual herbivore was alone. The results indicated that neither herbivore species influenced the survival of the other.

### Herbivore - predator interactions

The herbivores plus predator treatments reduced leaf area compared to the control (P = 0.02; Table 3). The presence of *C. carnea* did not influence the number of leaves on plants exposed to *G. calmariensis* (P = 0.05). The presence of predators affected survival of herbivores but had no effect on plant measurements. Over 20% of *G. calmariensis* survived in the *G. calmariensis* plus *C. carnea* treatment (Table 4), which is similar to the *G. calmariensis* alone treatment. When both herbivores were in the same cage with *C. carnea*, *G. calmariensis* survival was unaffected (Table 4). *G. calmariensis* survival in the *M. lythri* with both *G. calmariensis* and *H. axyridis* treatment was significantly less (12.4%) than in the *M. lythri *plus *G. calmariensis* treatment (29.6%) (Table 4). The lowest survival of *M. lythri* (2.2 per 12 leaves per plant) was observed in the *M. lythri* plus both *G. calmariensis* and *H. axyridis* treatment.

### Plant-predator interactions

At least one of the three predators released in each cage survived, indicating that predation occurred within the cages. At the end of the experiment, the predators were either in their pupal stage or last instar. The presence of predators did not affect any plant measurement (Table 3).

## Discussion

These findings have several implications for the understanding of multitrophic interactions associated with *L. salicaria*. First, the two herbivores (*G. calmariensis* and *M. lythri*) did not have an additive negative effect on *L. salicaria*. Second, two predatory species (*C. carnea* and *H. axyridis*) decreased *M. lythri* survival, and *H. axyridis* reduced *G. calmariensis* survival when *M. lythri* was present. Finally, predator presence did not indirectly benefit the plant, based upon the parameters measured.

Several studies have attempted to determine if multiple species of herbivores increase the success of weed biological control projects (Denoth et al. 2002). In the biological control program for the control of the invasive weed species, *Lantana camara* L., temporally separated natural enemies including (*Teleonemia scrupulosa* Stal (Hemiptera: Tingidae) during summer months and three species of Lepidoptera during winter months, contributed to the suppression of *L. camara* (Andres and Goeden, 1971). In some cases multiple herbivore species do not increase levels of biological control (Myers 1985; Hunt-Joshi and Blossey 2005a). In a 4-year field cage study, there was no increase in damage to *L. salicaria* when two spatially separated herbivores, *G. calmariensis* and a root feeding weevil, *Hylobius transversovittatus* Goeze (Coleoptera: Curculionidae), were present (Hunt-Joshi and Blossey 2004). By itself, *G. calmariensis* reduced *L. salicaria* height, reproductive ability, and aboveground biomass whereas *H.*  *transversovittatus* increased mortality of actively growing stems and thinned *L. salicaria* stands (Hunt-Joshi and Blossey 2004). However, in combined herbivore treatments, no increased suppression of *L*. *salicaria* was observed (Hunt-Joshi and Blossey 2004). In our study, an additive effect due to the herbivores *G*. *calmariensis* and *M. lythri* on *L*. *salicaria* was not observed because *M*. *lythri* alone did not reduce any of the plant characteristics assessed.

In the absence of predators, survival of one herbivore was not affected by the presence of the other herbivorous species. Previous studies examined coexistence of *G*. *calmariensis* and *G*. *pusilla* on *L*. *salicaria* (Blossey 1995) and interactions of the spatially separated root-feeding weevil, *H. transversovittatus*, and the herbivore, *G*. *calmariensis* on *L. salicaria* plants (Hunt-Joshi and Blossey 2005a). Although *G*. *pusilla* and *G*. *calmariensis* adults aggregate at the same sites and use the same host plant, these herbivorous species have similar competitive abilities and coexist (Blossey 1995). Root herbivory by *H. transversovittatus* did not affect *G*. *calmariensis*; in contrast, *G*. *calmariensis* herbivory negatively affected the survival of larvae of the root weevil *H. transversovittatus* (Hunt-Joshi and Blossey 2005a).

Although specialist predators and parasites are eliminated in the quarantine process before release (Harley and Forno 1992), naturally occurring generalist enemies in the release habitats have the potential to reduce establishment and success of the introduced herbivores (Goeden and Louda 1976). In our study species of generalist predators were chosen that occur in *L*. *salicaria*-infested wetlands (Sebolt and Landis 2004; Wiebe and Obrycki 2004). The results showed that survival of *G*. *calmariensis* in the treatment cages was not reduced by the presence of predators, except when *H. axyridis* and *M. lythri* were present. Previously, we showed that *G*. *calmariensis* was suitable for preimaginal development and survival of *C. carnea* and *H. axyridis* (Matos and Obrycki 2006). However, in the present study, *C. carnea* did not reduce *G*. *calmariensis* numbers compared to when *G*. *calmariensis* was alone. Neonate *G*. *calmariensis* feed within *L*. *salicaria* shoot tips presumably avoiding predation (Sebolt and Landis 2002). In the presence of the predator *Coleomegilla maculata* (De Geer) (Coleoptera: Coccinellidae), *G*. *calmariensis* neonate survival was higher in *L*. *salicaria* shoot tips (70%) than those neonates exposed on *L*. *salicaria* leaves (7.1%) (Sebolt and Landis 2002). A shift in behavior of herbivores or predators may alter the occurrence of a trophic cascade (Schmitz et al. 1997).

The suitability of prey for *H. axyridis* was higher for *M*. *lythri* compared to *G*. *calmariensis* larvae (Matos and Obrycki 2006). In the current study, it seems likely that *M. lythri* supplemented the low nutritional quality of *G*. *calmariensis* for *H. axyridis* development and survival. Similarly to *G*. *calmariensis*, *M*. *lythri* is found on leaves and stems and this may have created an opportunity for *H. axyridis* to attack *G*. *calmariensis.* Sebolt and Landis (2004) showed that the attack rates of *H. axyridis* on *G*. *calmariensis* first to third instars ranged from 60 to 100%. Our study showed higher *G*. *calmariensis* mortality when *M*. *lythri* was present and preyed upon by *H. axyridis* compared to the mortality when *H. axyridis* was absent. Because we did not include a treatment with only *G*. *calmariensis* plus *H. axyridis*, this treatment design did not determine whether the presence of *M. lythri* influenced predation of *G*. *calmariensis.*

*M. lythri* survival was significantly reduced in the presence of the predators. *M. lythri* did not reduce internode length; however, internode length was shorter in the *M*. *lythri* plus *H. axyridis* treatment than in the *M*. *lythri* alone treatment. Possibly *M*. *lythri* altered its behavior in the presence of *H. axyridis* and this behavior resulted in *M. lythri* moving to more protected sites where its feeding was detrimental to internode length.

*C*. *carnea* and *H. axyridis* did not indirectly benefit the growth of *L. salicaria.* Although *H. axyridis* caused *G*. *calmariensis* and *M. lythri* mortality and *C. carnea* caused *M. lythri* mortality, herbivory was not significantly reduced in this greenhouse cage study. During a 4-year field study, an opportunistic predator *Plagiognathus politis* (Hemiptera: Miridae) consumed large numbers of *G*. *calmariensis* eggs and young larvae (Hunt-Joshi et al. 2005b). However, after the second and third season, *G*. *calmariensis* populations increased to levels that caused significant defoliation (Hunt-Joshi et al. 2005b). Hunt-Joshi et al. (2005b) suggested that a more controlled experiment where predators and herbivore population levels were manipulated could result in a strong trophic cascade effect. Our study was a controlled short-term greenhouse experiment where herbivore and predator levels were manipulated, but no indirect positive effect of the two species of generalist predators on *L*. *salicaria* was found.
